# Supplementary material for: Prevalence, etiology, and transmission of fibropapillomatosis in Olive Ridley turtles at a mass-nesting colony in the Mexican Pacific
Source: PLoS One. 2026 Jan 7;21(1):e0339193. doi: 10.1371/journal.pone.0339193 (PMC12779068; doi:10.1371/journal.pone.0339193)
Supplement: S1 Table — Number of females per nesting season was estimated by dividing the average number of nests by the clutch frequency (using a mean value of 2.5 nests per female per season, according to Abreu-Grobois and Plotkin, 2008). (PDF) [file pone.0339193.s001.pdf]

S1 Table. Estimated number of nesting females during the past three nesting seasons at Playa Escobilla, Oaxaca, Mexico. Number of females per nesting season was estimated by dividing the average number of nests by the clutch frequency (using a mean value of 2.5 nests per female per season, according to Abreu-Grobois and Plotkin, 2008).

Nesting season	Estimated number of nests per season*	Estimated number of females per nesting season
2019-2020	980,750	392,300
2020-2021	1,250,885	500,354
2021-2022	1,327,944	531,178
**Mean**	**1,186,526**	**474,611**

*Nesting data were provided by the Comisión Nacional de Áreas Naturales Protegidas (CONANP)

Abreu-Grobois A, Plotkin P. (IUCN SSC Marine Turtle Specialist Group). *Lepidochelys olivacea*. The IUCN Red List of Threatened Species 2008: e.T11534A3292503. https://dx.doi.org/10.2305/IUCN.UK.2008.RLTS.T11534A3292503.en. Accessed on 19 October 2025.
