# Supplementary material for: Prevalence, etiology, and transmission of fibropapillomatosis in Olive Ridley turtles at a mass-nesting colony in the Mexican Pacific
Source: PLoS One. 2026 Jan 7;21(1):e0339193. doi: 10.1371/journal.pone.0339193 (PMC12779068; doi:10.1371/journal.pone.0339193)
Supplement: S2 Table — (PDF) [file pone.0339193.s002.pdf]

S2 Table. PCR Primers used for detection of the Chelonid alphaherpervirus 5 (ChAHV5)

Primer	Target gene	Primer sequence (5'-3')	Length fragment (bp)	Reference
UL18-F	Capsid protein	GTGGAACCCCGCCGGGTAAT	140	Alfaro-Nuñez and Gilbert 2014
UL18-R		TGATCCGGGCCGAGTAGCGG		Alfaro-Nuñez and Gilbert 2015
UL30-F	DNA polymerase	AGCATCATCCAGGCCCACAATCT	445	Lu et al. 2000
UL30-R		CGGCCAGTTCCGGCGCGTCGACCA		Lu et al. 2001
