# Supplementary material for: Prevalence, etiology, and transmission of fibropapillomatosis in Olive Ridley turtles at a mass-nesting colony in the Mexican Pacific
Source: PLoS One. 2026 Jan 7;21(1):e0339193. doi: 10.1371/journal.pone.0339193 (PMC12779068; doi:10.1371/journal.pone.0339193)
Supplement: S1 Fig — Sequences isolated from olive ridley turtles at Playa Escobilla are indicated in green (sample group is indicated by A = FP-affected turtles, B = healthy tissue of FP turtles, and C = clinically healthy turtles) and from Ozobranchus branchiatus leeches in blue. Bootstrap values (≥50%) are indicated at the respective nodes. The tree is rooted to Testudine alphaherpesvirus 3 (GenBank accession number NC027916). For sequences retrieved from GenBank, the accession numbers are provided in parentheses. Localities’ abbreviations are BR = Brazil, MX = Mexico, PR = Puerto Rico, U.S. = United States, WI = West Indies. (PDF) [file pone.0339193.s008.pdf]

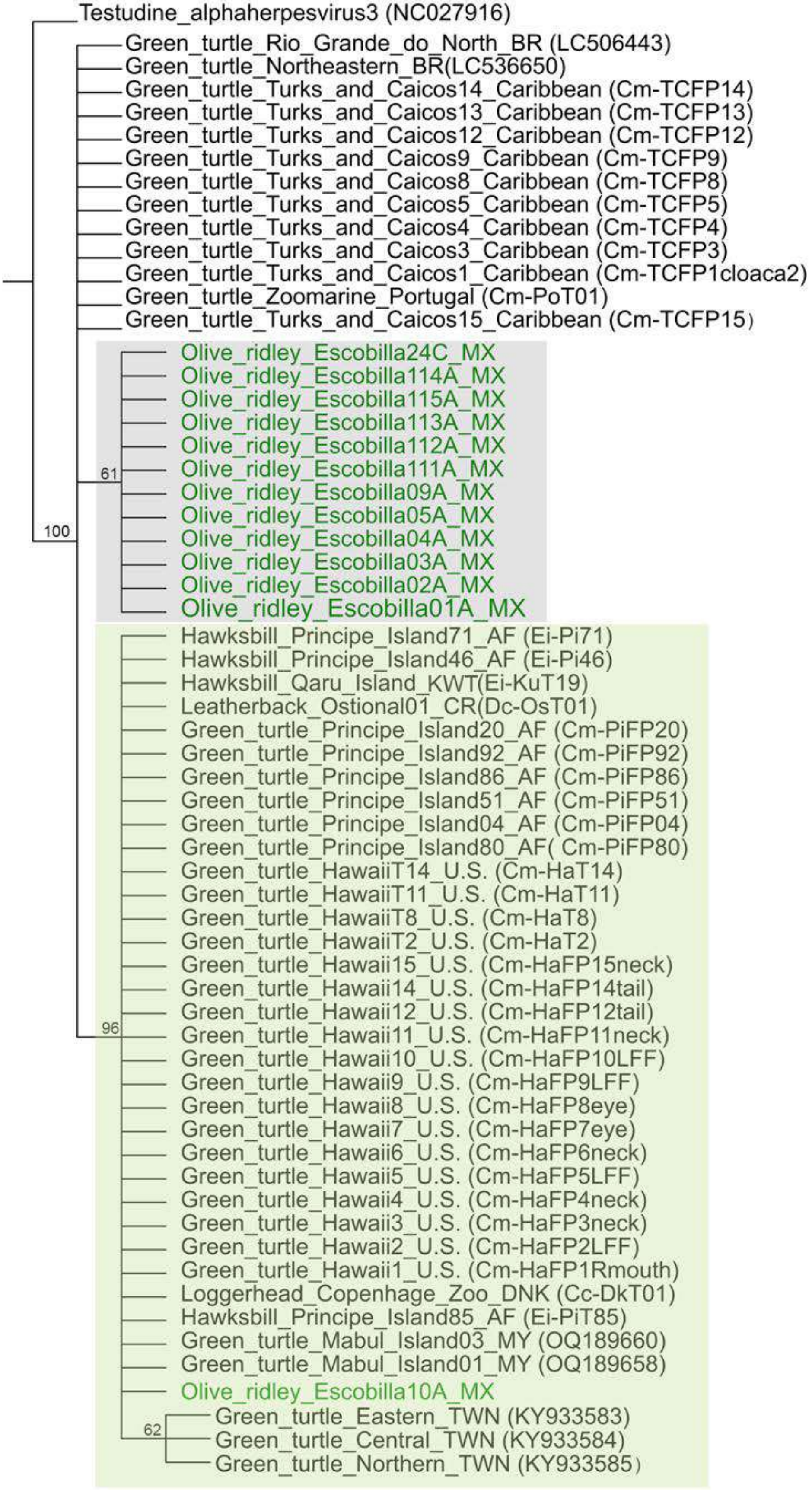
Testudine_alphaherpesvirus3 (NC027916)
Green_turtle_Rio_Grande_do_North_BR (LC506443)
Green_turtle_Northeastern_BR(LC536650)
Green_turtle_Turks_and_Caicos14_Caribbean (Cm-TCFP14)
Green_turtle_Turks_and_Caicos13_Caribbean (Cm-TCFP13)
Green_turtle_Turks_and_Caicos12_Caribbean (Cm-TCFP12)
Green_turtle_Turks_and_Caicos9_Caribbean (Cm-TCFP9)
Green_turtle_Turks_and_Caicos8_Caribbean (Cm-TCFP8)
Green_turtle_Turks_and_Caicos5_Caribbean (Cm-TCFP5)
Green_turtle_Turks_and_Caicos4_Caribbean (Cm-TCFP4)
Green_turtle_Turks_and_Caicos3_Caribbean (Cm-TCFP3)
Green_turtle_Turks_and_Caicos1_Caribbean (Cm-TCFP1cloaca2)
Green_turtle_Zoomarine_Portugal (Cm-PoT01)
Green_turtle_Turks_and_Caicos15_Caribbean (Cm-TCFP15)
Olive_ridley_Escobilla24C_MX
Olive_ridley_Escobilla114A_MX
Olive_ridley_Escobilla115A_MX
Olive_ridley_Escobilla113A_MX
Olive_ridley_Escobilla112A_MX
Olive_ridley_Escobilla111A_MX
61
Olive_ridley_Escobilla09A_MX
Olive_ridley_Escobilla05A_MX
100
Olive_ridley_Escobilla04A_MX
Olive_ridley_Escobilla03A_MX
Olive_ridley_Escobilla02A_MX
Olive_ridley_Escobilla01A_MX
Hawksbill_Principe_Island71_AF (Ei-Pi71)
Hawksbill_Principe_Island46_AF (Ei-Pi46)
Hawksbill_Qaru_Island_KWT(Ei-KuT19)
Leatherback_Ostional01_CR(Dc-OsT01)
Green_turtle_Principe_Island20_AF (Cm-PiFP20)
Green_turtle_Principe_Island92_AF (Cm-PiFP92)
Green_turtle_Principe_Island86_AF (Cm-PiFP86)
Green_turtle_Principe_Island51_AF (Cm-PiFP51)
Green_turtle_Principe_Island04_AF (Cm-PiFP04)
Green_turtle_Principe_Island80_AF(Cm-PiFP80)
Green_turtle_HawaiiT14_U.S. (Cm-HaT14)
Green_turtle_HawaiiT11_U.S. (Cm-HaT11)
Green_turtle_HawaiiT8_U.S. (Cm-HaT8)
Green_turtle_HawaiiT2_U.S. (Cm-HaT2)
Green_turtle_Hawaii15_U.S. (Cm-HaFP15neck)
Green_turtle_Hawaii14_U.S. (Cm-HaFP14tail)
Green_turtle_Hawaii12_U.S. (Cm-HaFP12tail)
96
Green_turtle_Hawaii11_U.S. (Cm-HaFP11neck)
Green_turtle_Hawaii10_U.S. (Cm-HaFP10LFF)
Green_turtle_Hawaii9_U.S. (Cm-HaFP9LFF)
Green_turtle_Hawaii8_U.S. (Cm-HaFP8eye)
Green_turtle_Hawaii7_U.S. (Cm-HaFP7eye)
Green_turtle_Hawaii6_U.S. (Cm-HaFP6neck)
Green_turtle_Hawaii5_U.S. (Cm-HaFP5LFF)
Green_turtle_Hawaii4_U.S. (Cm-HaFP4neck)
Green_turtle_Hawaii3_U.S. (Cm-HaFP3neck)
Green_turtle_Hawaii2_U.S. (Cm-HaFP2LFF)
Green_turtle_Hawaii1_U.S. (Cm-HaFP1Rmouth)
Loggerhead_Copenhage_Zoo_DNK (Cc-DkT01)
Hawksbill_Principe_Island85_AF (Ei-PiT85)
Green_turtle_Mabul_Island03_MY (OQ189660)
Green_turtle_Mabul_Island01_MY (OQ189658)
Olive_ridley_Escobilla10A_MX
Green_turtle_Eastern_TWN (KY933583)
62
Green_turtle_Central_TWN (KY933584)
Green_turtle_Northern_TWN (KY933585)
