# Supplementary figures and images for: Prevalence, etiology, and transmission of fibropapillomatosis in Olive Ridley turtles at a mass-nesting colony in the Mexican Pacific
Source: PLoS One. 2026 Jan 7;21(1):e0339193. doi: 10.1371/journal.pone.0339193 (PMC12779068; doi:10.1371/journal.pone.0339193)

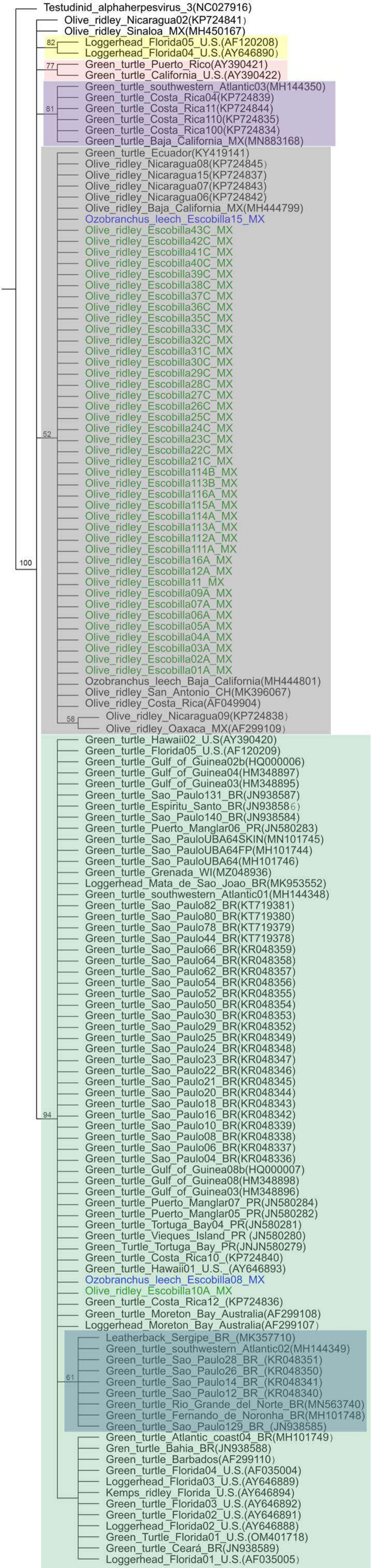

Supplement: S2 Fig — Sequences obtained from olive ridley turtles at Playa Escobilla are indicated in green (sample group is indicated by A = FP-affected turtles and C = clinically healthy turtles). Bootstrap values (≥50%) are indicated at the respective nodes. The tree is rooted with Testudine alphaherpesvirus 3 (GenBank accession number NC027916). For sequences retrieved from GenBank or the Dryad repository, the accession numbers or ID are provided in parentheses. Localities’ abbreviations are AF = Africa, BR = Brazil, CR = Costa Rica, DNK = Denmark, KWT = Kuwait, MY = Malaysia, MX = Mexico, TWN = Taiwan, U.S. = United States. (PDF) [file pone.0339193.s009.pdf]
