# Supplementary material for: Prevalence, etiology, and transmission of fibropapillomatosis in Olive Ridley turtles at a mass-nesting colony in the Mexican Pacific
Source: PLoS One. 2026 Jan 7;21(1):e0339193. doi: 10.1371/journal.pone.0339193 (PMC12779068; doi:10.1371/journal.pone.0339193)
Supplement: Attachment — Submitted filename: Labastida_ChAHV5_Response to Reviewers_NOV.docx [file pone.0339193.s011.docx]

RESPONSES TO REVIEWERS

Manuscript reference: **PONE-D-25-37483**

Prevalence, etiology, and transmission of fibropapillomatosis in Olive Ridley turtles at a mass-nesting colony in the Mexican Pacific

Luisa Maria Diele-Viegas, Ph. D.
Academic Editor
PLOS ONE

Please find in this letter our responses to the reviewers’ comments. All changes in the manuscript that correspond to responses to Reviewer # 1 are in green type, those to Reviewer #2 are in red type. Additionally, responses to the Journal requirements are highlighted in purple. If you have any questions, please feel free to contact us for additional explanation.

**Journal Requirements**:

When submitting your revision, we need you to address these additional requirements.

1. Please ensure that your manuscript meets PLOS ONE's style requirements, including those for file naming.

Yes, we verify that our manuscript meets all style and file naming requirements.

2. To comply with PLOS One submissions requirements, in your Methods section, please provide additional information regarding the experiments involving animals and ensure you have included details on (1) methods of sacrifice, (2) methods of anesthesia and/or analgesia, and (3) efforts to alleviate suffering.

Yes, we confirm that our manuscript meets these requirements. In addition to the statements regarding legal permits for sampling activities (Lines 181-185 in the revised version of the manuscript), we included information on the efforts made to minimize discomfort and stress to the individuals, as well the sanitization measures applied to reduce the risk of infection from the tissue sampling (Lines 232-235 in the revised version of the manuscript). In our study, no animals were sacrificed or injured to the degree that required anesthesia or analgesia.

3. Thank you for stating in your Funding Statement: [This project was partially funded by the Programa de Apoyo a Proyectos de Investigación e Innovación Tecnológica IN215722 of the Universidad Nacional Autónoma de México (PAPIIT-UNAM). The Dirección General de Asuntos del Personal Académico of the Universidad Nacional Autónoma de México (DGAPA-UNAM) provided financial support to E.L.E. through a postdoctoral fellowship. The Secretaría de Ciencias, Humanidades, Tecnología e Innovación (SECIHTI) supplied a grant scholarship (No. 812903) to K.M.L.T. (No. CVU: 1165342) and financial support through the Investigadoras e Investigadores por México program to V.I.V. (No. project 538).].

Please provide an amended statement that declares *all* the funding or sources of support (whether external or internal to your organization) received during this study, as detailed online in our guide for authors at <http://journals.plos.org/plosone/s/submit-now>.  Please also include the statement “There was no additional external funding received for this study.” in your updated Funding Statement.

Please include your amended Funding Statement within your cover letter. We will change the online submission form on your behalf.

We made changes to the Funding Statement and provided an amended version with the requested details.

4. Thank you for stating the following financial disclosure: [This project was partially funded by the Programa de Apoyo a Proyectos de Investigación e Innovación Tecnológica IN215722 of the Universidad Nacional Autónoma de México (PAPIIT-UNAM). The Dirección General de Asuntos del Personal Académico of the Universidad Nacional Autónoma de México (DGAPA-UNAM) provided financial support to E.L.E. through a postdoctoral fellowship. The Secretaría de Ciencias, Humanidades, Tecnología e Innovación (SECIHTI) supplied a grant scholarship (No. 812903) to K.M.L.T. (No. CVU: 1165342) and financial support through the Investigadoras e Investigadores por México program to V.I.V. (No. project 538).].

Please state what role the funders took in the study.  If the funders had no role, please state: ""The funders had no role in study design, data collection and analysis, decision to publish, or preparation of the manuscript.""

If this statement is not correct you must amend it as needed.

Please include this amended Role of Funder statement in your cover letter; we will change the online submission form on your behalf.

We made changes to the Funding Statement and provided an amended version with the requested details.

5. We note that the grant information you provided in the ‘Funding Information’ and ‘Financial Disclosure’ sections do not match.

This project was partially funded by the Programa de Apoyo a Proyectos de Investigación e Innovación Tecnológica IN215722 of the Universidad Nacional Autónoma de México (PAPIIT-UNAM). The Dirección General de Asuntos del Personal Académico of the Universidad Nacional Autónoma de México (DGAPA-UNAM) provided financial support to E.L.E. through a postdoctoral fellowship. The Secretaría de Ciencias, Humanidades, Tecnología e Innovación (SECIHTI) supplied a grant scholarship (No. 812903) to K.M.L.T. (No. CVU: 1165342) and financial support through the Investigadoras e Investigadores por México program to V.I.V. (No. project 538).

When you resubmit, please ensure that you provide the correct grant numbers for the awards you received for your study in the ‘Funding Information’ section.

We have made the requested changes

6. When completing the data availability statement of the submission form, you indicated that you will make your data available on acceptance. We strongly recommend all authors decide on a data sharing plan before acceptance, as the process can be lengthy and hold up publication timelines. Please note that, though access restrictions are acceptable now, your entire data will need to be made freely accessible if your manuscript is accepted for publication. This policy applies to all data except where public deposition would breach compliance with the protocol approved by your research ethics board. If you are unable to adhere to our open data policy, please kindly revise your statement to explain your reasoning and we will seek the editor's input on an exemption. Please be assured that, once you have provided your new statement, the assessment of your exemption will not hold up the peer review process.

All genetic sequences generated in this study have been deposited in the DNA Data Bank of Japan (DDBJ) under accession numbers LC899718–LC899775 and LC899376–LC899383. These sequences will be publicly available through the DDBJ website within 2 to 7 days from November 4, 2025, once released by DDBJ. The Data Availability Statement in the manuscript has been updated accordingly to reflect this information.

7. We note that Figure 4 in your submission contain [map/satellite] images which may be copyrighted. All PLOS content is published under the Creative Commons Attribution License (CC BY 4.0), which means that the manuscript, images, and Supporting Information files will be freely available online, and any third party is permitted to access, download, copy, distribute, and use these materials in any way, even commercially, with proper attribution. For these reasons, we cannot publish previously copyrighted maps or satellite images created using proprietary data, such as Google software (Google Maps, Street View, and Earth). For more information, see our copyright guidelines: <http://journals.plos.org/plosone/s/licenses-and-copyright>.

We require you to either (1) present written permission from the copyright holder to publish these figures specifically under the CC BY 4.0 license, or (2) remove the figures from your submission:

1. You may seek permission from the original copyright holder of Figure 4 to publish the content specifically under the CC BY 4.0 license.

We recommend that you contact the original copyright holder with the Content Permission Form (<http://journals.plos.org/plosone/s/file?id=7c09/content-permission-form.pdf>) and the following text:

“I request permission for the open-access journal PLOS ONE to publish XXX under the Creative Commons Attribution License (CCAL) CC BY 4.0 (<http://creativecommons.org/licenses/by/4.0/>). Please be aware that this license allows unrestricted use and distribution, even commercially, by third parties. Please reply and provide explicit written permission to publish XXX under a CC BY license and complete the attached form.”

Please upload the completed Content Permission Form or other proof of granted permissions as an ""Other"" file with your submission.

In the figure caption of the copyrighted figure, please include the following text: “Reprinted from [ref] under a CC BY license, with permission from [name of publisher], original copyright [original copyright year].”

2. If you are unable to obtain permission from the original copyright holder to publish these figures under the CC BY 4.0 license or if the copyright holder’s requirements are incompatible with the CC BY 4.0 license, please either i) remove the figure or ii) supply a replacement figure that complies with the CC BY 4.0 license. Please check copyright information on all replacement figures and update the figure caption with source information. If applicable, please specify in the figure caption text when a figure is similar but not identical to the original image and is therefore for illustrative purposes only.

The following resources for replacing copyrighted map figures may be helpful:

USGS National Map Viewer (public domain): <http://viewer.nationalmap.gov/viewer/>

The Gateway to Astronaut Photography of Earth (public domain): <http://eol.jsc.nasa.gov/sseop/clickmap/>

Maps at the CIA (public domain): <https://www.cia.gov/library/publications/the-world-factbook/index.html> and <https://www.cia.gov/library/publications/cia-maps-publications/index.html>

NASA Earth Observatory (public domain): <http://earthobservatory.nasa.gov/>

Landsat: <http://landsat.visibleearth.nasa.gov/>

USGS EROS (Earth Resources Observatory and Science (EROS) Center) (public domain): [http://eros.usgs.gov/#](http://eros.usgs.gov/)

Natural Earth (public domain): <http://www.naturalearthdata.com/>

Figure 4 was created specifically for this study and is therefore not subject to copyright restrictions. The map was produced using QGIS v.3.16 (free and open-source software). The base layer was obtained from OpenStreetMap contributors (available from https://www.openstreetmap.org), licensed under the Open Database License (ODbL; https://www.openstreetmap.org/copyright), and accessed through the FAO Geospatial Platform (https://data.apps.fao.org/). The figure caption now includes proper attribution to OpenStreetMap and clarifies that the data are available under the ODbL license, as well as in Acknowledgments section (Lines 469-471 and 649-6532 in the revised version of the manuscript).

8. If the reviewer comments include a recommendation to cite specific previously published works, please review and evaluate these publications to determine whether they are relevant and should be cited. There is no requirement to cite these works unless the editor has indicated otherwise.

This comment does not apply to our case

9. Please review your reference list to ensure that it is complete and correct. If you have cited papers that have been retracted, please include the rationale for doing so in the manuscript text, or remove these references and replace them with relevant current references. Any changes to the reference list should be mentioned in the rebuttal letter that accompanies your revised manuscript. If you need to cite a retracted article, indicate the article’s retracted status in the References list and also include a citation and full reference for the retraction notice.

We have thoroughly reviewed the reference list and would like to note the following:

We did not find any retracted articles in our reference list.

We removed outdated references and those not strictly necessary, as suggested by Reviewer #1. As a result, the reference numbers have changed in this revised version of the manuscript.

**Review Comments to the Author**

Please use the space provided to explain your answers to the questions above. You may also include additional comments for the author, including concerns about dual publication, research ethics, or publication ethics. (Please upload your review as an attachment if it exceeds 20,000 characters)

**Reviewer #1**

The article entitled “Prevalence, etiology, and transmission of fibropapillomatosis in Olive Ridley turtles at a mass-nesting colony in the Mexican Pacific” is well-written and presents a sound methodology. The study successfully addressed all its objectives, which is essential for a high-quality scientific paper.

1. (A) My main concern with the manuscript is the use of a pie chart, which could be replaced with a bar chart, for instance, to improve clarity. (B) Additionally, the reference list includes a large number of sources published more than ten years ago and relatively few from the past five years. I recommend that the authors revise the bibliography and consider removing outdated references that are not strictly necessary.

Comment 1A. We appreciate Reviewer #1 suggestion to replace the pie chart in Figure 4 with a bar chart. Nonetheless, pie charts are widely used to illustrate haplotype composition and genetic variant proportions in population studies. We explored the use of a bar chart for Figure 4, but it was less effective in conveying the data and did not improve interpretability; therefore, we opted to retain the pie chart format.

Comment 1B. We removed outdated references and those not strictly necessary, as suggested. As a result, the reference numbers have changed in this revised version of the manuscript.

Finally, I congratulate the authors on this valuable contribution to sea turtle conservation.

**Reviewer #2**

The manuscript is well-written, with an appropriate methodology and a solid discussion. However, some methodological points need to be detailed, and a few parts of the discussion could be improved.

**ABSTRACT**

1. The abstract is excellent; it just needs a final concluding sentence to summarize the main finding.

We have included a final sentence summarizing our key findings (Lines 43-46 in the revised version of the manuscript).

**INTRODUCTION**
2. The introduction is a bit long, but I would not change it because it contains a lot of interesting information that helps the reader understand the study's scope. In lines 148 to 153, it would be beneficial to add the prevalence of the disease in the mentioned locations, so the reader can understand whether the prevalence is high or low in this species.

We have now included the FP prevalence data corresponding to the nesting beaches mentioned in lines 148–153 original version of MS (Lines 151-156 in the revised version of the manuscript).

**MATERIAL AND METHODS**

The Materials and Methods section is good but requires more detail in some parts.

3. In lines 189-192, please cite Table 1, which contains the estimated population size. I felt the absence of this information at this point.

In lines 189–192, we clarified that the sampling effort was estimated based on the population size from the three previous nesting seasons (2019–2020, 2020-2021, and 2021-2022), whereas Table 1 presents the estimated female population size per arribada for the nesting season during this study (2022–2023). To fully address the reviewer’s request, we have now included this data in a supplementary table (Table S1, Line 197 in the revised version of the manuscript).

4. Please explain in more detail how the beach was monitored during collections. Was it monitored every day? What times were the collections made? Was the patrol done on foot or with a vehicle (e.g., ATV)? How many people participated in the monitoring?

Night surveys were conducted every day along the nesting beach to detect FP cases in nesting females. Surveys were carried out on foot along the nesting area during the first five nights of each *arribada*, between 21:00 and 04:00 h. During these nights, we walked approximately 7 km, covering the main nesting zone. However, when nesting activity was concentrated in high-density areas, efforts were focused on those zones. Each survey was conducted by a team of four to five people, most of whom were volunteers. On some nights when volunteers were unavailable, only two or three people participated. Additional methodological details are provided in the Materials and Methods section (Lines 197-201 in the revised version of the manuscript).

5. What specific non-toxic paint was used? Were the animals also tagged, or were they only identified with the paint?

Tagging with Inconel tags is a widely used method that provides valuable information on sea turtle populations and, in this case, could have yielded useful data on FP-affected individuals. Unfortunately, our budget for this project was limited, and the cost of Inconel tags exceeded our available financial resources. Therefore, all females examined were tagged using non-toxic, water-based traffic paint (Ecolatex®, Lines 205-206 in the revised version of the manuscript). In this context, a recent study by Saladin and Freggi (2024) suggested that the use of Inconel tags may pose a risk of cross-contamination with ChAHV5 through infected tagging equipment. Although this study is not conclusive, it highlights the importance of implementing biosecure measures to prevent the indirect transmission of ChAHV5 during tagging activities.

Saladin C, Freggi D. The tale of the sea turtle flipper tagging technique, a global environmental impact assessment. Wildl Lett. 2024. 2: 44-59.

6. From what part of the body was the tissue sample obtained (e.g., neck, flipper)?

The tissue collected was obtained from the flipper. We have completed this information in the manuscript (Lines 226-227 in the revised version of the manuscript).

7. How was the blood collected? Was it during or after nesting? Was an anticoagulant used? In what type of tube was the blood stored after collection?

Sampling was conducted after oviposition to avoid interference with nesting activities. Blood samples were collected from the dorsal cervical sinus following the protocol described by Dutton (1996). Approximately 0.5 mL of blood was obtained from each individual and preserved in 96% ethanol at 4 °C in sterile Eppendorf tubes. Blood samples were collected without the use of anticoagulants. We have now included this information in the manuscript (Lines 229-232 in the revised version of the manuscript).

8. How were the tumors collected? Was the entire tumor collected, or just a piece? Was there a preference for smaller tumors, which the literature suggests have a higher viral load?

Tumor samples were obtained using a sterile 6 mm dermal punch. Each sample measured approximately 30 mm² and weighed about 140 mg; only a portion of the tumor was collected. We selected tumors larger than 1-3 cm in length and sampled tissue from the peripheral region, which apparently helps to minimize bleeding. Complete tumor excision was not performed to avoid potential injury or infection. We also reviewed literature for studies examining the correlation between viral load and tumor size but found none. We would therefore appreciate any references the reviewers could suggest, to consider this aspect in future studies.

9. What is the prevalence of leeches on the turtles? Can you estimate this, or at least indicate if they are commonly found on turtles or if they are rare? This information would be interesting to the discussion (e.g., viral transmission).

*Ozobranchus branchiatus* is a common ectoparasite of olive ridley sea turtles. In a study conducted in Michoacán and Oaxaca (including Playa Escobilla), Mexico, a prevalence of 39% was reported among 28 turtles examined. That study also recorded 85 individuals of *O. branchiatus* on 11 turtle hosts (Gámez-Vivaldo et al., 2006). In our study, leeches were found on 37 turtles, with 3–10 individuals per host. In three olive ridley females, however, we observed large aggregations of more than 100 leeches, particularly in the neck region.

Gámez-Vivaldo S, Osorio-Sarabia D, Peñaflorers-Salazar C, García HJL, Ramírez LJ. Identification of parasites and epibionts in the olive ridley turtle (*Lepidochelys olivacea*) that arrived to the beaches of Michoacan and Oaxaca, Mexico. Vet Mex. 2006, 37:431-440.

10.Was only one leech collected from each animal, or were more than one collected?

In our study, leeches were found on 37 turtles, with 3–10 individuals per host, from which we collected 3–5 leeches. In three olive ridley females, however, we observed large aggregations of more than 100 leeches, particularly in the neck region; from these turtles, 56–70 individuals were collected. For molecular analyses, one leech per turtle was processed, assuming that ChAHV5 circulates systemically in the host’s blood (viremia). Therefore, all leeches collected could potentially test positive for ChAHV5 due to their hematophagous feeding behavior.

**RESULTS**
Congratulations on the large sample size of turtles examined. The effort you made is commendable, even with a large population in the area.

11. In lines 320-321, it says 22 tissue samples, with 9 being blood and 14 being tissue (9 + 14 = 23). Please double-check this information.

We corrected the number of analyzed samples and double-checked the following: 18 tumor samples, 2 healthy flipper tissue samples from FP-affected turtles, 21 samples from healthy turtles —including 12 flipper tissue samples and 9 blood samples— and one muscle tissue sample from a healthy turtle, for a total of 42 samples (Lines 344-348 in the revised version of the manuscript).

**DISCUSSION**
The discussion needs some adjustments.

12. Line 451: What was the sample size of the 1988 study? Including this information is important to understand how many animals were sampled and to be able to confidently state that the prevalence has decreased.

We have included the sample sizes analyzed in Vasconcelos et al. (1998) and in our study to better contextualize the FP prevalence estimates across these two studies (Lines 482-484 in the revised version of the manuscript).

13. Lines 453-455: Was the site monitored before this? It would be good to have information on how long this population has been monitored to be able to state that there were no cases before then.

Population records of olive ridley turtles at Playa Escobilla —as well as in surrounding areas of the Mexican Pacific— date back to the late 1960s, when commercial exploitation for turtle skins to be used in the leather industry began. Because of this overexploitation, monitoring of olive ridley turtles at Playa Escobilla started in 1967, with the establishment of the first Sea Turtle (Tortuguero) Camp dedicated to research, protection, and conservation of sea turtles along the Oaxaca coast. Continuous records of the olive ridley nesting population have been maintained on this beach since 1973. Based on these extensive monitoring data, we can state that, before 1980, no cases of FP were reported in olive ridley turtles from Playa Escobilla, as such cases would have been documented or mentioned in historical records.

Peralta BE, Luna T. Tortuga Golfina. In Gaona PO, Barragán R. editors. Las tortugas marinas en México: logros y perspectivas para su conservación. México: PROCER-CONANP; México: 2016. pp. 81-102.

Comisión Nacional de Áreas Naturales Protegidas (CONANP). Plan de Manejo Santuario Playa Escobilla. Secretaría de Medio Ambiente y Recursos Naturales (SEMARNAT). México: 2024. pp. 14-19.

14. Lines 464-468: The comparison made here is unfair, as it is known that green sea turtles are the most affected species and therefore have the highest prevalence. Additionally, juveniles also have a much higher prevalence than adults. Either remove this part of the discussion or compare it with other nesting areas of the olive ridley turtle. If you don't find many studies on olive ridleys, try comparing them with other species, but from nesting areas, not foraging areas. Only then will you be able to support the information in lines 470-472.

As suggested by the reviewer, we have deleted the previous comparison and included a new one using available data from Ostional, Costa Rica, which is also a mass-nesting beach. We believe that this change provides stronger support for this part of the discussion (Lines 495-501 in the revised version of the manuscript).

15. Lines 479-480: Are the green sea turtles mentioned here juveniles? If so, it is more relevant to compare them with adults of the same species or, if there are no articles available, with adults of other species.

We have changed this sentence but retained the mention of eye tumors reported in juvenile green turtles to contextualize the case of an eye tumor found in an olive ridley turtle in this study (Lines 510-513 in the revised version of the manuscript)

16. Lines 487-490: Again, are the green sea turtles juveniles? It's always good to make this information clear because it makes a big difference. The discussion would be much better and interesting if it compared between species in nesting areas.

We have adjusted this section and added a comparison using available data from a solitary nesting beach in Baja California, Mexico (Lines 520-529 in the revised version of the manuscript).

17. Lines 501-502: What are the "other tissues" (2 of 2)?

We have clarified that we are referring to biopsies of flipper from FP-affected females (Line 534 in the new version of the manuscript).

18. Line 507: There is a typo: "god" standard should be "good" standard.

We have corrected this typo (Lines 539-540 in the new version of the manuscript).

19. Topic "viral DNA detection in nesting olive ridley females": What do these findings reveal, considering the prevalence of fibropapillomatosis found? Even with a low sample size of tissue from healthy turtles, the prevalence of the virus was high. Could this be related to the environment they live in or the animals' health?

A key finding of our study was the high prevalence of ChAHV5 in turtles without physical signs associated with FP. This result is consistent with previous studies conducted in several sea turtle species (Alfaro-Núñez et al., 2014; Espinoza et al., 2020; Zamana et al., 2021). These findings provide important insights into the pathogenesis of ChAHV5, which remains only partially understood. Herpesviruses, including ChAHV5 have a biphasic life cycle that includes a primary infection followed by a latent phase. In the manuscript (Lines 553-556 in the revised version of the manuscript), we highlight that during latency, the viral genome persists in the nucleus of host cells while limiting the expression of viral proteins. However, ChAHV5 can reactivate when the immune response is reduced due to environmental factors, stress, or poor health conditions. In this sense, comprehensive reviews on FP have emphasized its multifactorial nature, suggesting that the FP is more accurately described as a syndrome rather than a single disease (Lines 553-556 in the revised version of the manuscript Dujon et al., 2018; Saladin and Freggi, 2024).

Alfaro-Núñez A, Frost Bertelsen M, Bojesen AM, Rasmussen I, Zepeda-Mendoza L, Tange Olsen M, et al. Global distribution of Chelonid fibropapilloma-associated herpesvirus among clinically healthy sea turtles. BMC Evol Bio. 2014;14: 1-12

Dujon AM, Schofield G, Venegas RM, Thomas F, Ujvari B. Sea turtles in the cancer risk landscape: a global meta-analysis of fibropapillomatosis prevalence and associated risk factors. Pathogens. 2021;10: 1295.

Espinoza J, Hernández E, Lara-Uc MM, Reséndiz E, Alfaro-Núñez A., Hori-Oshima S, Medina-Basulto G. Genetic analysis of Chelonid herpesvirus 5 in marine turtles from Baja California Peninsula. EcoHealth. 2020;17: 258-263.

Zamana RR, Gattamorta MA, Cruz-Ochoa PF, Navas‐Suárez PE, Sacristán C, Rossi S, et al. High occurrence of chelonid alphaherpesvirus 5 (ChHV5) in green sea turtles *Chelonia mydas* with and without fibropapillomatosis in feeding areas of the São Paulo Coast, Brazil. J Aquat Anim Health. 2021;33: 252-263.

Saladin C, Freggi D. The tale of the sea turtle flipper tagging technique, a global environmental impact assessment. Wildl Lett. 2024. 2: 44-59.

20. Line 536: Which turtle species and in which location was the study conducted that found 90% viral detection in leeches?

We have completed the requested information in the manuscript (Line 570 in the revised version of the manuscript)

21. Could the low viral detection also be related to the small number of leeches sampled?

We consider that, rather than the small number of leeches sampled, the method used to detect the ChAHV5 UL30 gene is the most critical factor influencing the capacity to detect viral presence in *Ozobranchus* leeches. In the studies Farrell et al. (2021) and by Rittenburg et al. (2021) quantitative PCR (qPCR) was used for detection and quantification of the ChAHV5 UL30 gene. For pathogen detection, qPCR offers greater sensitivity and specificity (detection limit: 1–10 copies per cell) compared to conventional PCR (detection range: 10³–10⁴ copies per cell), and low viral loads could therefore result in false negatives when using conventional PCR.

Farrell JA, Yetsko K, Whitmore L, Whilde J, Eastman CB, Ramia DR, et al. Environmental DNA monitoring of oncogenic viral shedding and genomic profiling of sea turtle fibropapillomatosis reveals unusual viral dynamics. Commun Biol. 2021;4: 565

Rittenburg LR, Kelley JR, Mansfield KL, Savage AE. Marine leech parasitism of sea turtles varies across host species, seasons, and the tumor disease fibropapillomatosis. Dis Aquat Organ. 2021;143: 1-12.

22. I missed a final concluding paragraph summarizing the main findings.

We added a concluding paragraph to summarize the main findings (Lines 622-636 in the revised version of the manuscript)
